# Effectiveness of peer pressure on computed tomography use for dizziness/vertigo patients

**DOI:** 10.1097/MD.0000000000014887

**Published:** 2019-03-15

**Authors:** Po-Chun Chuang, Yi-Syun Huang, Charng-Yen Chiang, E-Wai Zhang, Fu-Jen Cheng

**Affiliations:** a Department of Emergency Medicine, Kaohsiung Chang Gung Memorial Hospital, Niao-Sung, Kaohsiung; b Chang Gung University College of Medicine, Guishan District, Taoyuan City, Taiwan.

**Keywords:** dizziness, emergency, hospital, peer influence, vertigo

## Abstract

Dizziness/vertigo is a common complaint in the emergency department (ED). We aimed to evaluate the effect of peer pressure on decision making in emergency physicians (EPs) to use computed tomography (CT) for patients with dizziness/vertigo.

We conducted a before-and-after retrospective case review of patients who visited the ED with dizziness/vertigo. EPs were categorized into 3 groups according to seniority (in years of experience: >12, 7–12, and <7). The rate of CT use for EPs, patient number, and CT use were e-mailed monthly to update the EP team on the benchmark rate and shape of the behavior.

Among the 1657 (preintervention) and 1508 (postintervention) patients with dizziness/vertigo, 320 (19.3%) and 230 (15.3%), respectively, underwent brain CT. A decrease in the rate of CT use was observed in the postintervention group (odds ratio [OR] = 0.743, 95% confidence interval [CI] = 0.615–0.897), especially in junior EPs (years of experience, <7; OR = 0.667, 95% CI: 0.474–0.933) and younger patients (age, <60) (OR = 0.625, 95% CI: 0.453–0.857).

The intervention strategy created peer pressure through e-mail reminders and decreased the rate of CT use for patients with isolated dizziness/vertigo, especially in junior EPs and younger patients.

## 1 Introduction

Dizziness, a common complaint in the emergency department (ED), accounts for 2.5% of all ED visits in the United States.^[1]^ Although the most common causes of dizziness/vertigo are benign, a potentially serious underlying disease, such as cerebellar or brain stem stroke, may be overlooked.^[2,3]^

As a consequence of the uncertainty and cost of a misdiagnosis, emergency physicians (EPs) may reduce the testing threshold for brain imaging in managing these low-probability, high-morbidity situations. Unnecessary head computed tomography (CT) examination may lead to an increased length of ED stay,^[4]^ medical costs, and radiation exposure (a potential carcinogen).^[5,6]^ The use of CT imaging to examine patients presenting with dizziness has increased tremendously, from 9.4% to 37.4% in the United States between 1995 and 2009.^[7]^

A previous study demonstrated that EPs vary in their respective decisions to either admit or discharge general ED patients. Senior EPs were found to have lower discharge rates compared with their junior colleagues.^[8]^ Another study found support for the effectiveness of peer pressure on changing disposition decisions made by EPs.^[9]^ This study used a behavior modifying measure by the creation of team norms updated by monthly e-mail reminders. Norms are the rules that the team agrees to follow and designate a standard for average performance by the whole team. Once developed, team norms are used to guide and shape team members’ behavior. We created a “team norm” imposed peer-pressure effect by announcing the CT use rate of each EP through monthly e-mail reminders. The purpose of this study was thus to evaluate the peer-pressure effect on the decisions of CT use for dizziness/vertigo patients by EPs with varying seniority.

## 2 Methods

Ethical approval (number 201600764B0C10) was obtained for this study from the Ethics Committee of Kaohsiung Chang Gung Memorial Hospital.

### 2.1 Study design

To evaluate the effectiveness of peer pressure on changing EP decisions concerning CT use for dizziness/vertigo patients, we conducted a before-and-after retrospective case review of patients who visited the ED. This study intervention created a “team norm” that imposed an unspoken peer pressure effect by announcing the CT-use rate of each EP. The CT-use rate of each EP was calculated and announced by e-mail in the middle of the following month to enhance the team norm and shape the behavior of each EP. E-mails included the detailed numbers of patient visits of dizziness/vertigo and CT use for each shift handled by each EP. Grading and listing of the CT-use rate data for all EPs was also included, and the top 3 (including most CT use and least CT use) EPs were highlighted. Only statistical figures were reported to the EPs without any additional rewards or punishments. E-mail reminders for this investigation were initially sent in July 2016. The preintervention study period spanned from March 1, 2016, to July 31, 2016, and the postintervention period was from September 1, 2016 to January 31, 2017. The study was approved by our hospital's institutional review board and has been performed in accordance with the ethical standards as set forth in the 1964 Declaration of Helsinki and its later amendments. For this type of study, formal consent from subjects was not required.

### 2.2 Setting and population

This study was conducted in a tertiary academic medical center in Southern Taiwan with over 2500 acute beds and an average of 72,000 adult ED visits per year. The medical records of nontraumatic patients who were older than 17 years of age and visited the ED with a principal diagnosis of dizziness and vertigo were extracted from the ED administrative database using the International Classifications of Diseases Tenth Revision coding system (dizziness, code R42 and vertigo, code H81.3). Electronic charts were reviewed to identify patients with isolated dizziness/vertigo. Such cases were defined as individuals who presented with a primary complaint of dizziness or vertigo, were screened by ED clinicians, and did not have any documented evidence of recent onset neurologic findings. Patients with documented recent onset abnormal neurologic findings, including cranial nerve examination, cerebellar function tests, or muscle power or sensory change, were excluded. We defined dizziness/vertigo with a central nervous system (CNS) origin by a new finding on brain CT images that could explain the dizziness/vertigo. The analysis was further confirmed by brain magnetic resonance image (MRI) or diagnosed as such by a neurologist at hospital discharge.

The EPs were categorized into 3 groups according to seniority. Group “>V12” consisted of 10 senior physicians with more than 12 years of work experience. Group “V7-V12” consisted of 9 physicians with 7 to 12 years of work experience (intermediate seniority). Group “<V7” consisted of 10 junior physicians with <7 years of work experience.

Our EPs were all trained with a 4-year emergency medicine residency conducted by the Taiwan Society of Emergency Medicine in a qualified teaching hospital. None of the physicians that were included in this study had been deposed in a lawsuit as a defendant during the prior 5 years. In our ED, residents help to evaluate patients, but the EPs make the final decision regarding CT examination scheduling and admission. EPs were paid according to the number of shifts worked and not the number of patients treated; therefore, test ordering was not motivated by profit.

### 2.3 Variables and outcome measures

Age, sex, triage level, and risk factors for ischemic stroke including hypertension, diabetes, previous transient ischemic attack/stroke, coronary artery disease, hypercholesterolemia, atrial fibrillation, current smoker, and alcoholism were collected from the medical record charts. Patient disposition, ED length of stay (LOS), and final discharge diagnosis of the CNS origin dizziness/vertigo by a neurologist were also documented. The primary outcome was brain CT use during ED evaluation, and the secondary outcome was ED LOS and hospital admission.

### 2.4 Data analyses

The results of the descriptive analyses of independent variables are reported as percentages or mean ± standard deviations. Independent variables were analyzed using Chi-squared, Mann–Whitney *U*, and Student *t* tests. The relationship of seniority with CT use and hospital admission was analyzed using the Chi-squared test, and logistic regression was used to obtain the odds ratio (OR), 95% confidence interval (CI), and *P*-value for trends. A *P*-value <.05 was regarded as statistically significant. SPSS version 18.0 (SPSS Inc, Chicago, IL) was used for all statistical analyses.

## 3 Results

### 3.1 Characteristics of study subjects (patients and physicians)

During the study period, a total of 73,360 patients visited the ED, of whom 3356 (4.57%) had dizziness/vertigo as their primary diagnosis. Among the patients with dizziness/vertigo as their primary diagnosis, 132 patients were excluded because they were diagnosed by a corresponding author, including both “preintervention” and “postintervention” groups to avoid experimenter effects. About 59 cases were excluded because of a chart-documented new neurologic deficit.

Our study group comprised the remaining 3165 patients; 1657 and 1508 were enrolled in preintervention and postintervention groups, respectively. Patients were assessed by the 29 EPs in our department. The median number of patients assessed by each EP was 58 and 53 in preintervention and postintervention groups, respectively.

An bivariate analysis revealed no significant difference between preintervention and postintervention groups in age (*P* = .908), sex (*P* = .205), patients with hypertension (*P* = .276), coronary artery disease (*P* = .453), diabetes (*P* = .354), previous transient ischemic attack/stroke (*P* = .818), current smoker (*P* = .306), hypercholesterolemia (*P* = .940), atrial fibrillation (*P* = .378), heavy alcohol consumption (*P* = .536), triage status (*P* = .246), mean arterial pressure during triage (*P* = .949), admission (*P* = .934), 72 hours revisit for vertigo (*P* = .211), or final diagnosis of dizziness/vertigo with CNS origin (*P* = .559). Among the total 69 patients of 72 hours revisits, 7 had a final diagnosis of CNS origin. Three patients of 72 hours revisits with a final diagnosis of central origin belonged to the preintervention group; the other 4 patients belonged to the postintervention group.

Furthermore, 320 and 230 (19.3% and 15.3%, respectively; *P* = .003) patients received brain CT examinations in each group. The CT-use rate decreased in the postintervention group (19.3% vs 15.3%, *P* = .003). Patients had longer ED LOS in the postintervention group (5.4 ± 8.2 vs 6.0 ± 8.3), but the difference was not statistically significant (Table 1).

**Table 1:**
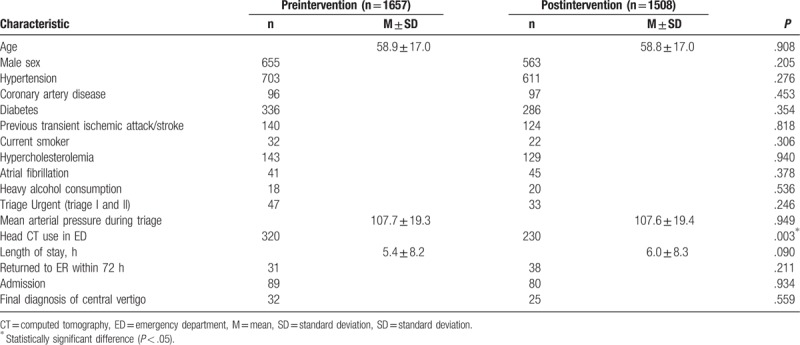
Demographic characteristics of emergency department patients in the preintervention and postintervention groups.

Of the 57 (1.80%) patients who received a final diagnosis of a disease of CNS origin by a neurologist during discharge, 15 patients had a posterior circulation ischemic lesion including vertebrobasilar insufficiency; 22 patients had a hemisphere ischemic lesion; 8 patients had hemorrhagic lesions, including subdural hemorrhage and cerebellar hemorrhage; 11 patients had brain tumor, including benign and malignant tumor; and 1 patient had vasculitis.

### 3.2 Association between patient and physician characteristics and decision making

As shown in Table 2, bivariate analysis revealed that EPs tended to order brain CTs in older (*P* < .001) patients, as well as those with hypertension (*P* < .001), diabetes (*P* = .001), previous transient ischemic attack/stroke (*P* < .001), hypercholesterolemia (*P* = .033), atrial fibrillation (*P* = .001), triage urgent (*P* < .001), and a higher mean arterial pressure at triage (*P* < .001).

**Table 2:**
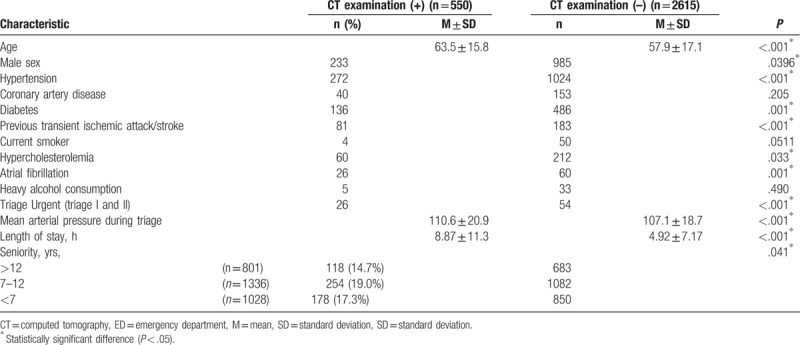
Demographic characteristics of the emergency department patients in the brain computed tomography (+) or computed tomography (–) groups.

**Figure 1:**
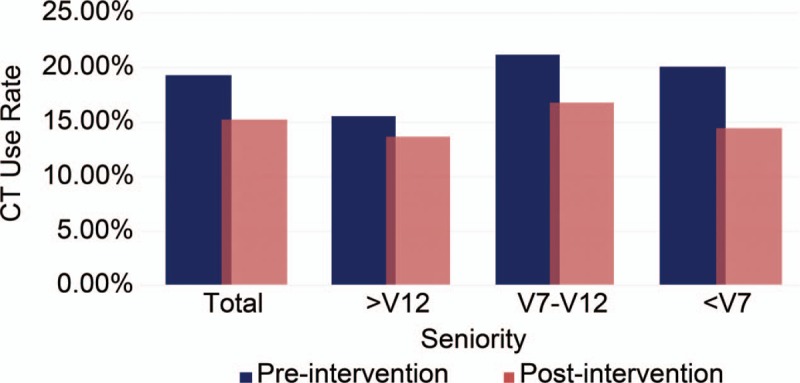
Computed tomography use rate in preintervention and postintervention.

Figure 1 shows CT use rate in the preintervention and postintervention groups. In the postintervention group, CT use rate decreased in all EP groups, especially in the V7-12 and <V7 groups.

### 3.3 Association between patient and physician characteristics and intervention

Table 3 shows the number and percentage of patients who received CT examination in the preintervention and postintervention groups at different age levels treated by each EP group. Decreased CT-use rate was found in the postintervention group treated by intermediate (V7-12) and junior (<V7) EPs (*P* = .041 and .015, respectively), as well as in the postintervention group for patients <60 years old (*P* = .002).

**Table 3:**
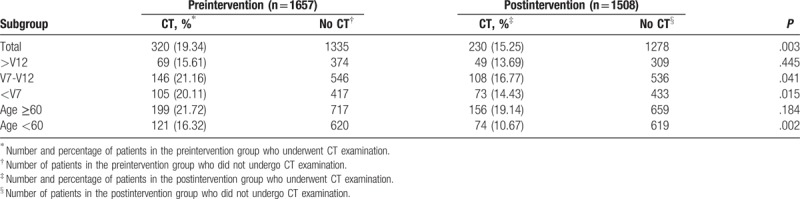
Computed tomography (CT) performed at the emergency department.

**Figure 2:**
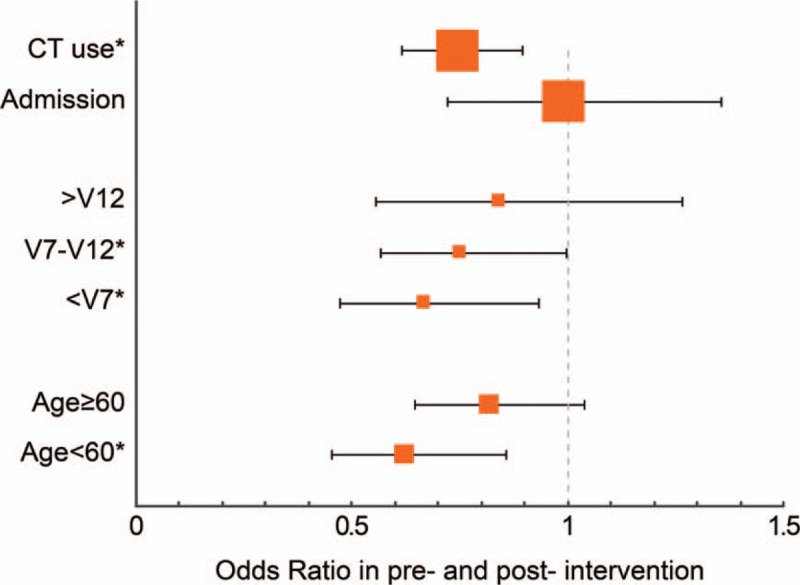
Comparison of the adjusted odds ratio of head computed tomography (CT) use and admission. The confounders that the model was adjusted for age, male sex, hypertension, diabetes, previous transient ischemic attack/stroke, hypercholesterolemia, atrial fibrillation, and mean blood pressure during triage.

We subsequently performed the analysis to adjust for confounding factors. The following patient and clinical characteristics confounded the relationship between EP groups and CT use: age, male sex, hypertension, diabetes, previous transient ischemic attack/stroke, hypercholesterolemia, atrial fibrillation, and mean blood pressure during triage. In the adjusted analysis, after controlling for patient- and visit-level variables, there was a significant association between intervention and head CT use in different age groups and EP groups. The adjusted OR of head CT use was 0.743 (95% CI = 0.615–0.897) in the postintervention group compared with preintervention group (Fig. 2). Compared with preintervention group, the adjusted OR of head CT use was 0.842 and 0.753 (CI: 0.567–0.998 and 0.474–0.933, respectively) in the postintervention group treated by intermediate (V7-12) and junior (<V7) EPs, respectively. Intervention and head CT use were not significantly associated in the senior EPs (>V12) after adjustment for patient-level confounding factors (OR = 0.842; CI: 0.556–1.266).

The adjusted odds of head CT use was 0.625 (CI: 0.453–0.857) in younger patients (<60 years old) of the postintervention group compared with the preintervention group. However, intervention was not associated with head CT use for older patients (age ≥ 60 years).

## 4 Discussion

Only a small percentage of patients who present with dizziness in the ED receive a final diagnosis of CNS-originated vertigo. A previous study indicated that the diagnostic rate of CNS-origin dizziness was 0.7% in patients without neurologic signs who were older than 44 years of age.^[3]^ Another study further found that 0.6% patients with isolated vertigo were diagnosed as dizziness/vertigo of CNS origin. Despite the low diagnostic rate of CT for vertigo patients, the proportion of patients presenting to the ED with dizziness who underwent a CT scan increased 169% from 1995 to 2004,^[1]^ whereas the proportion of patients who received a CNS-related diagnosis dropped by 62% during the same period.^[4]^ An additional study demonstrated increasing CT and MRI use with a rising annual cost in the US EDs.^[7]^ In 2011, the proportion of neuroimaging use in the ED for patients with dizziness was estimated to be 39.9% (39.4% CT, 2.3% MRI) with an average cost $1004. One study showed a 48% CT-use rate with a 0.74% diagnostic rate and an average cost of $1220 per CT scan.^[10]^ Some investigations have tried to identify neurologic examinations aside from CT to detect central vertigo, such as the head impulse test^[11,12]^; however, no evidence showed that these neurologic examinations can reduce CT used in ED, and no well-established guideline was introduced or accepted. The present study found that intervention resulted in an overall 4.06% (19.34% vs 15.25%, *P* = .003) decrease in CT use. Our ED treats approximately 4000 isolated dizziness/vertigo patients per year; a 4.06% decrease in CT use is therefore equivalent to 162.4 fewer CT scans and an annual cost savings of $198,128 in the US ED. This may be an effective way to decrease unnecessary neuroimaging and cost in ED.

In the present study, we employed 1 e-mail reminder per month as the feedback intervention. Isolated dizziness/vertigo cases in our ED reached around 300 per month, comprising an average of 10.7 cases per month, per physician. E-mail provided a relatively easy method by which to remind EPs of CT use and resulted in fewer scans; however, further studies might be needed to establish a standard method and time to administer audits and feedback in different situations.

The EPs vary significantly in their use of head CT.^[13]^ One previous study has demonstrated that EPs vary in their admit/discharge decision making for general ED patients. Most senior EPs have the lowest discharge rates.^[11]^ Another study showed that senior EPs have lowest mortality and fewest 72-hour returns. This superior quality of care is accompanied with a slightly longer LOS.^[14]^ Our study also showed EPs vary in their CT use for dizziness/vertigo patients. The most senior EPs (>12 years of experience) have a low CT ordering rate (14.7%), while a higher CT-use rate is seen in intermediate EPs (≥7 and ≤12 years of experience, 19.0%) and junior EPs (<7 years of experience, 17.3%). Furthermore, the CT use rate of senior EPs decreased after intervention (15.61–13.69%), but the difference was not statistically significant. These results suggest that peer pressure has less influence on senior EPs. One possible reason was that senior EPs differed in their clinical practice with junior and intermediate EPs.^[11,14]^ Second, senior EPs might have more experience and might relatively resist to changes in their clinical practice. Third, the CT use rate was relatively lower for senior EPs in the preintervention group, and it might be the reason the decreased CT use rate did not achieve statistical significance.

### 4.1 Limitations

This study has several limitations. First, the retrospective nature and relatively small sample number of EPs at a single teaching hospital may limit the implications and generalizability of our conclusions to other ED settings.

Second, the relatively small number of patients with CNS diagnoses may have limited the statistical power of between-group analyses. It is possible that some patients with a primary diagnosis of dizziness/vertigo were not considered by our review, because the inclusion criterion applied here utilized the International Classifications of Diseases Tenth Revision coding system (dizziness, code R42 and vertigo, code H81.3). If a physician did not use this code, those cases would not have been included.

Third, the present study was conducted in a single country. Other countries have diverse laws and regulations such that an EP's behavior may differ according to possible medical disputes. Fourth, this study was conducted in a country where national health insurance penetration and baseline CT utilization are high, while medical costs are relatively low. Our findings may not be necessarily applicable to countries in which health insurance penetration or baseline CT use is low, and medical costs are relatively high.

## 5 Conclusion

The intervention strategy presented herein applied peer pressure through e-mail reminders. Our results featured a decrease in CT use for patients with isolated dizziness/vertigo, particularly among junior EPs and in younger patients. Our method may effectively decrease CT use and unnecessary medical costs in ED.

## Author contributions

**Conceptualization:** Fu-Jen Cheng.

**Data curation:** Po-Chun Chuang, Yi-Syun Huang, Charng-Yen Chiang, E-Wai Zhang.

**Formal analysis:** Fu-Jen Cheng.

**Methodology:** Fu-Jen Cheng.

**Project administration:** Fu-Jen Cheng.

**Resources:** Fu-Jen Cheng.

**Software:** Fu-Jen Cheng.

**Supervision:** Fu-Jen Cheng.

**Validation:** Fu-Jen Cheng.

**Writing – original draft:** Po-Chun Chuang.

**Writing – review & editing:** Po-Chun Chuang.

Fu-Jen Cheng orcid: 0000-0003-1960-2274.
